# Differentiating Phenotypes of Coronavirus Disease-2019 Pneumonia by Electric Impedance Tomography

**DOI:** 10.3389/fmed.2022.747570

**Published:** 2022-05-19

**Authors:** András Lovas, Rongqing Chen, Tamás Molnár, Balázs Benyó, Ákos Szlávecz, Fatime Hawchar, Sabine Krüger-Ziolek, Knut Möller

**Affiliations:** ^1^Department of Anesthesiology and Intensive Therapy, Kiskunhalas Semmelweis Hospital, Kiskunhalas, Hungary; ^2^Institute of Technical Medicine, Furtwangen University, VS-Schwenningen, Germany; ^3^Department of Anesthesiology and Intensive Therapy, University of Szeged, Szeged, Hungary; ^4^Department of Control Engineering and Information Technology, Budapest University of Technology and Economics, Budapest, Hungary

**Keywords:** acute lung injury, compliance, Coronavirus-COVID-19, electric impedance tomography, recruitment maneuver

## Abstract

**Introduction:**

Coronavirus disease-2019 (COVID-19) pneumonia has different phenotypes. Selecting the patient individualized and optimal respirator settings for the ventilated patient is a challenging process. Electric impedance tomography (EIT) is a real-time, radiation-free functional imaging technique that can aid clinicians in differentiating the “low” (L-) and “high” (H-) phenotypes of COVID-19 pneumonia described previously.

**Methods:**

Two patients (“A” and “B”) underwent a stepwise positive end-expiratory pressure (PEEP) recruitment by 3 cmH_2_O of steps from PEEP 10 to 25 and back to 10 cmH_2_O during a pressure control ventilation of 15 cmH_2_O. Recruitment maneuvers were performed under continuous EIT recording on a daily basis until patients required controlled ventilation mode.

**Results:**

Patients “A” and “B” had a 7- and 12-day long trial, respectively. At the daily baseline, patient “A” had significantly higher compliance: mean ± *SD* = 53 ± 7 *vs*. 38 ± 5 ml/cmH_2_O (*p* < 0.001) and a significantly higher physiological dead space according to the Bohr–Enghoff equation than patient “B”: mean ± *SD* = 52 ± 4 *vs*. 45 ± 6% (*p* = 0.018). Following recruitment maneuvers, patient “A” had a significantly higher cumulative collapse ratio detected by EIT than patient “B”: mean ± *SD* = 0.40 ± 0.08 *vs*. 0.29 ± 0.08 (*p* = 0.007). In patient “A,” there was a significant linear regression between the cumulative collapse ratios at the end of the recruitment maneuvers (*R*^2^ = 0.824, *p* = 0.005) by moving forward in days, while not for patient “B” (*R*^2^ = 0.329, *p* = 0.5).

**Conclusion:**

Patient “B” was recognized as H-phenotype with high elastance, low compliance, higher recruitability, and low ventilation-to-perfusion ratio; meanwhile patient “A” was identified as the L-phenotype with low elastance, high compliance, and lower recruitability. Observation by EIT was not just able to differentiate the two phenotypes, but it also could follow the transition from L- to H-type within patient “A.”

**Clinical Trial Registration:**

www.ClinicalTrials.gov, identifier: NCT04360837.

## Introduction

Severe acute respiratory syndrome Coronavirus-2 (SARS-CoV-2)-associated pneumonia can deteriorate into acute respiratory distress syndrome (ARDS). However, severe Coronavirus disease-2019 (COVID-19) pneumonia fulfills the Berlin criteria of ARDS (1), this type of acute lung injury is exceptionally specific. The SARS-CoV-2 acquired ARDS is characterized by near normal respiratory mechanics associated with hypoxemia in almost half of the cases (2). Even more a significant dissociation was observed as distinct lung mechanics were detailed with the same level of grievous oxygenation disturbance. This perception led to the distinction of two phenotypes of COVID-19 pneumonia by Gattinoni et al. (3) whereas, low (L-) type is featured by lower elastance and almost normal compliance (>50 ml/cmH_2_O), low ventilation-to-perfusion ratio (V_A_/Q), lower recruitability, and estimated lung weight. On the contrary, high (H-) type is characterized by higher elastance and low compliance (<40 ml/cmH_2_O), high right-to-left shunt, higher recruitability, and lung weight. Of note, L-type can transit into H-type by advancing time.

The two different phenotypes require differing mechanical ventilation and therapeutic approach. According to the recommendations, due to the various characteristics in pathophysiology, H-type can profit from the standard settings for ARDS: lower tidal volume, higher positive end-expiratory pressure (PEEP) level and prone positioning. While H-type benefits from an excursive management: more permissive tidal volume, lower PEEP setting and applying prone positioning just as a rescue therapy (3).

The patient individualized approach can be challenging for the attending physician taking care of the COVID-19 pneumonia patients. Even more, as the clinical state of the critically ill patient is fluctuating and phenotype can alter while the leading symptom, the hypoxia is equally profound. However, bedside measurement implemented by the ventilator supports the follow-up, the gold standard of ARDS diagnostics is still an imaging technique, the computed tomography (CT) (4). However, the CT scan of the chest is at high resolution and it gives information not just about the complete lungs but about all organs situated in the thorax, CT examination performed on a daily basis is not feasible. Meanwhile, electrical impedance tomography (EIT) is a radiation-free functional imaging technique providing continuous information about the lungs at the bedside (5). EIT is capable of estimating not just lung aeration, but also the ratio of collapse and overdistension during a PEEP trial (6).

Our objective was to investigate the different phenotypes of COVID-19 pneumonia patients under a stepwise PEEP incremental and decremental recruitment trial performed on a daily basis under continuous EIT monitoring to estimate if impedance tomography is capable of differentiating the various phenotypes of COVID-19 pneumonia.

## Materials and Methods

### Study Registration

The study was approved by the Human Investigation Review Board of the University of Szeged. The trial was registered in a public registry under the registration number NCT04360837 on ClinicalTrials.gov. Informed consent was obtained from the legal representatives of the patients.

### Study Population

All patients admitted to the COVID-19 intensive care unit (ICU) of the University of Szeged, diagnosed with SARS-CoV-2 pneumonia, following a positive polymerase chain reaction were considered for investigation during the first wave of the pandemic in Hungary. Further inclusion criteria were orotracheal intubation and pressure-controlled ventilation mode at a sedation level of minimum-4 on the Richmond Agitation Sedation Scale (RASS). Exclusion criteria were age under 18, pregnancy, pulmonectomy, and lung resection in the past medical history, clinically end-stage chronic obstructive pulmonary disease, severe hemodynamic instability with vasopressor refractory shock, severe bullous emphysema, and chest drainage *in situ* due to pneumothorax and/or bronchopleural fistula. During the first national surge of the epidemic, seven patients were admitted to the ICU. Out of them, one patient was not intubated, two patients were lightly sedated at RASS-3 level and were ventilated in a pressure support mode, one of them was excluded because of severe bullous emphysema and one of them because of end-stage chronic obstructive pulmonary disease regarding to the exclusion criteria of the investigation. Finally, two patients could undergo the research protocol, patient “A” on 7 and patient “B” on 12 consecutive days.

### Experimental Protocol

Following orotracheal intubation and initiation of deep sedation to at least RASS-4 with continuous intravenous infusion of propofol and sufentanil patients were ventilated in pressure-controlled mode with a tidal volume of 6 ml/kg. Patients underwent a once-daily PEEP incremental and decremental recruitment maneuver until controlled ventilation mode was required according to their clinical stage. During the repeated interventions pressure-controlled ventilation mode was applied with a constant pressure control of 15 cmH_2_O by a Mindray SV300 respirator (Mindray Bio-Medical Electronics Co., Shenzen, China). The fraction of inspired oxygen (FiO_2_) and respiratory rate were set according to the discretion of the attending physician. Pending the incremental limb PEEP was increased in 3 cmH_2_O steps from basal PEEP 10 to top 25, reaching a peak pressure of 40 cmH_2_O. On the descending limb, PEEP was decreased in 3 cmH_2_O steps back to the initial level of 10. Each PEEP step were kept constant for 2 min and at each level, an inspiratory hold maneuver was performed to detect plateau pressure and static compliance (C_stat_). Vital parameters and volumetric capnography measurements of the ventilator were recorded throughout the intervention. Arterial blood gas samplings were performed at basal and at terminating PEEP levels of 10. Physiological dead space (V_D_) representing the sum of the anatomical plus the alveolar dead space was calculated by the Bohr–Enghoff equation.

Electrical impedance tomography measurements were recorded continuously during the increasing and decreasing limb of the PEEP recruitment maneuver by the Dräger PulmoVista 500 impedance tomography (Dräger Medical, Lübeck, Germany). The device has 16 electrodes equidistantly placed on the chest circumference in a transverse plane between the 5^th^ and 6^th^ intercostal space. EIT monitoring data were measured with adjacent injection current and adjacent voltage measurement with 50 frames per second. Time difference EIT images were reconstructed using the Newton–Raphson algorithm. It is demonstrated that the regional tidal volume correlates well with the pixel-wise conductivity variation (ΔZ) indicated by the EIT tidal image (7). Hence, the pixel compliance, required for the evaluation of global overdistension and collapse, can be calculated as:


(1) $${\text{Compliance}}_{pixel}=\frac{\Delta Z}{{\text{P}}_{plateau}-\text{PEEP}}$$

The complete estimation of collapse and overdistension was presented previously (8). Optimal PEEP can be determined by EIT (5) which was established for both patients. During the decremental PEEP trial phase, regional lung hyper distension and collapse ratios were estimated at each step. The crossover point between the curves of the decreasing line of overdistension and the increasing line of collapse indicated the optimal PEEP where the level of the two opposing factors was meeting.

### Statistical Analysis

For statistical analysis, Sigmaplot 14 (Systat Software Inc., San Jose, CA, USA) was used. Following the Shapiro–Wilk normality test data between two groups were tested by the *t*-test or the Mann–Whitney rank sum test, matched data were tested by the paired *t*-test or the Wilcoxon signed-rank test in a group. For multiple-comparison one-way repeated measures analysis of variance (ANOVA) with the Brown–Forsythe equal variance test, for comparison vs. a control group Bonferroni *t*-test or Dunnett's method was used. For regression analysis, simple linear regression was applied. The *p*-value was considered significant if <0.05.

## Results

### Patient Characteristics

**Figure 1:**
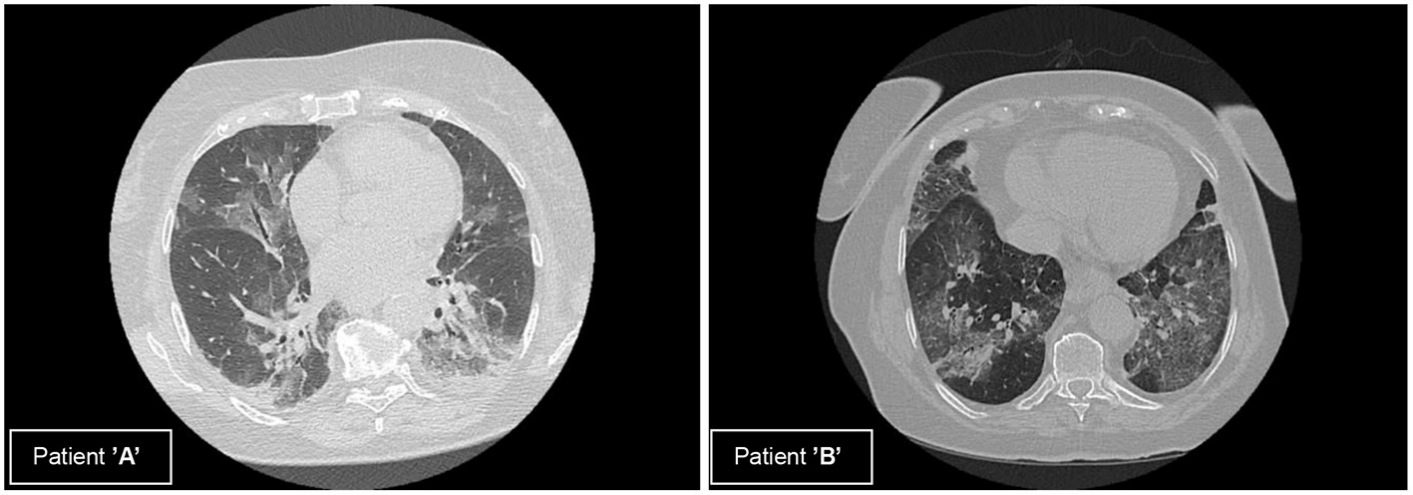
Computed tomography (CT) scans of patients **(A)** and **(B)**. **(A)** Multifocal, subpleural, bilateral ground glass opacities with subpleural traction in the dorsal regions. **(B)** Multifocal, subpleural, bilateral ground glass opacities, crazy paving dominantly on the left side, consolidation in the right basal region.

Patient “A” was ventilated for 7 days and patient “B” was ventilated for 12 days in a controlled mode. Both of them underwent a PEEP intervention on a daily basis (Table 1). CT scans of the chest were recorded right before the admission to ICU (Figure 1).

**Table 1:** Patient characteristics.

	**Patient “A”**	**Patient “B”**
Age (years)	67	81
Sex	Female	Female
Body-mass index (kg/m^2^)	30	31
APACHE II score	17	18
PEEP trials (N)	7	12
Comorbidities	Hypertension, Hypothyroidism	Hypertension
Days of symptomes before intubation	8	2
Hours of hospitalization before intubation	18	6
PaO_2_/FiO_2_ at inclusion (mmHg)	258	142
Outcome on ICU	Died	Died

*APACHE II, acute physiology and chronic health evaluation II; PEEP, positive end-expiratory pressure; ICU, intensive care unit*.

### Respiratory Mechanics

**Figure 2:**
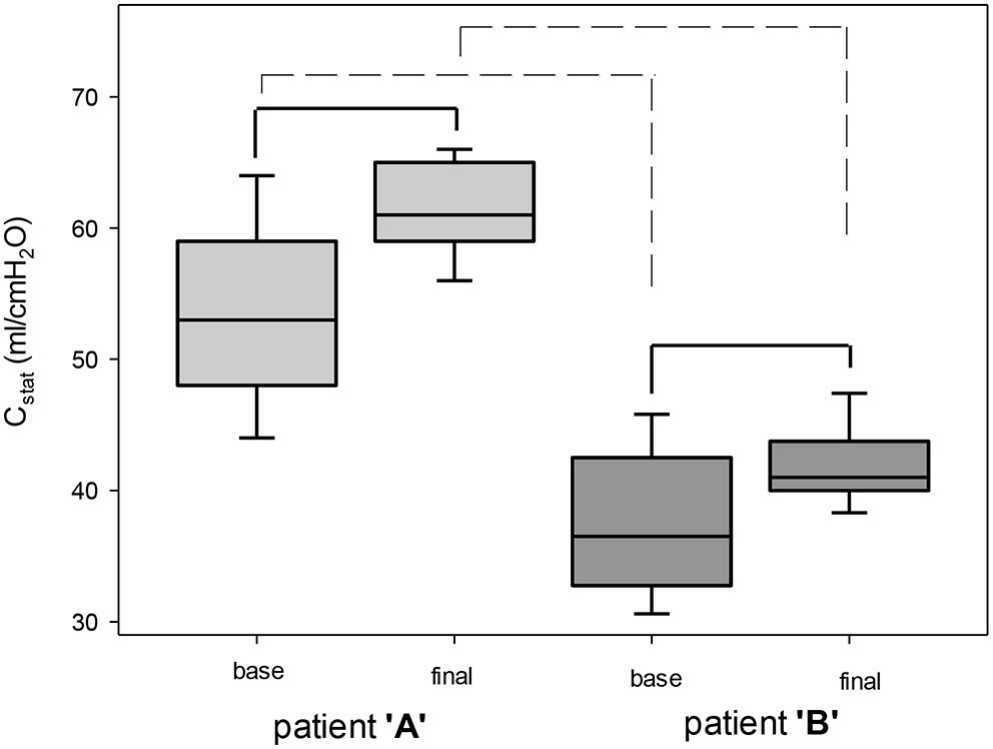
Compliance at baseline and following positive end-expiratory pressure (PEEP) trial. C_stat_, compliance. Solid line, significant difference within a patient. Dashed line, significant difference between patients. Box plots represent mean, ± *SD* and 5^th^-95^th^ percentile, *p* < 0.05.

**Figure 3:**
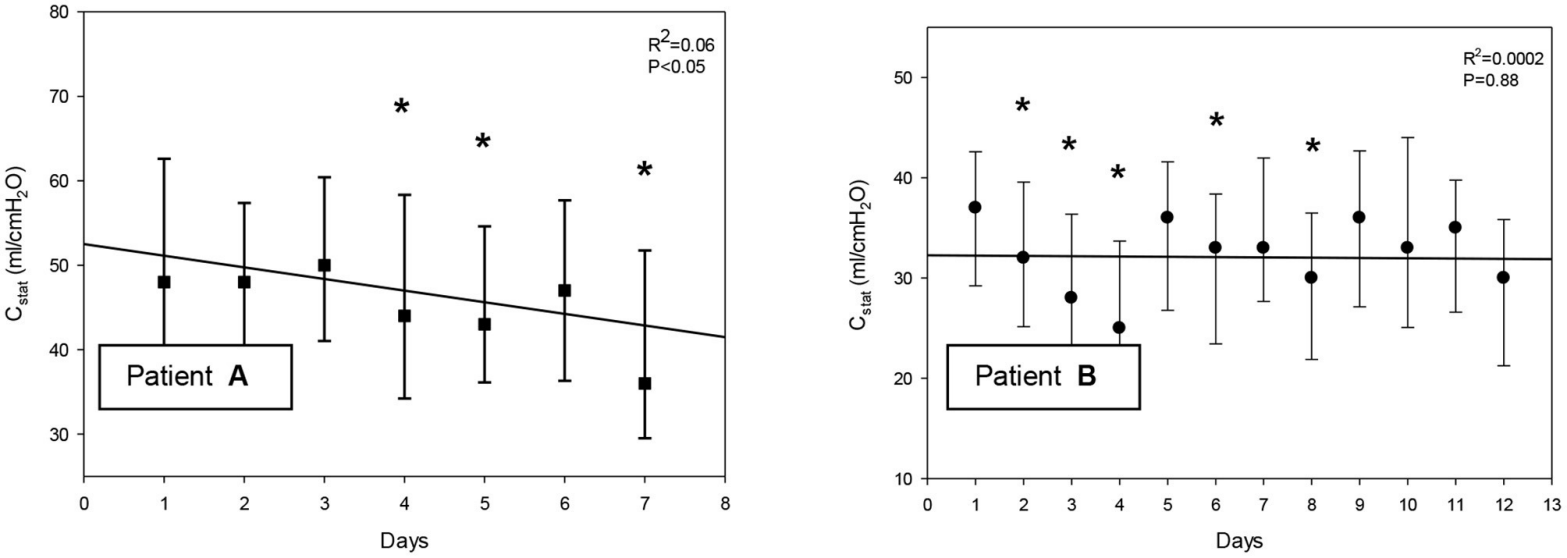
**(A,B)** Alteration in compliance by moving forward in days. C_stat_, compliance. Bars represent mean and ± *SD*. *significant difference as compared to day 1, *p* < 0.05.

At the daily baseline and following the intervention patient “A” had significantly higher C_stat_ than patient “B”. Following the PEEP recruitment trial C_stat_ significantly improved both with patients “A” and “B” (Figure 2). On days 4, 5, and 7 C_stat_ was significantly lower as compared to day 1 with patient “A.” With patient “B” C_stat_ was significantly lower on days 2, 3, 4, 6, 8, and 12 as compared to day 1. There was significant decreasing linear regression in C_stat_ with patient “A” by moving forward in days but not for patient “B” (Figure 3).

### Blood Gas and Capnography

With patient “A,” pH significantly increased and partial pressure of carbon dioxide in the arterial blood (P_a_CO_2_) significantly decreased following the recruitment maneuver. In the meantime, a not significant decrease in pH and a not significant increase in P_a_CO_2_ were detected with patient “B.” There was no significant alteration in partial pressure of oxygen in the arterial blood (P_a_O_2_)/FiO_2_, oxygen saturation (SO_2_), base excess (BE), and lactate with neither of the patients (Table 2).

**Table 2:** The average of blood gas results and physiological dead space ratios at the initial (base) and at the terminating (final) positive end-expiratory pressure (PEEP) levels of the daily trials.

	**Patient “A”**		**Patient “B”**	
	**Base**	**Final**	**Base**	**Final**
pH	7.45 ± 0.02	7.46 ± 0.02^a^	7.40 ± 0.05^b^	7.38 ± 0.04^b^
PaCO_2_ (mmHg)	44 ± 4	42 ± 4^a^	40 ± 5	40 ± 4
PaO_2_/FiO_2_	166 ± 34	184 ± 37	120 ± 30^b^	124 ± 26^b^
SO_2_ (%)	97 ± 2	97 ± 1	95 ± 2	95 ± 2^b^
BE	5.1 ± 2.4	4.7 ± 2.7	−1.3 ± 2.5^b^	−1.5 ± 2.6^b^
Lactate (mmol/L)	1.2 ± 0.2	1.3 ± 0.3	1.7 ± 0.9	1.7 ± 0.8
V_D_	0.53 [0.48–0.56]	0.47[0.44–0.50]^a^	0.45 ± 0.06^b^	0.47 ± 0.09
EtCO_2_ (mmHg)	39 ± 5	37 ± 4	36 ± 3	37 ± 3

*PaCO_2_, partial pressure of carbon dioxide in the arterial blood; PaO_2_/FiO_2_, partial pressure of oxygen in the arterial blood to fraction of inspired oxygen; SO_2_, oxygen saturation; BE, base excess; V_D_, physiological dead space; EtCO_2_, end-tidal carbon dioxide*,

a *significant difference within a patient*,

b *significant difference as compared to patient “A”, P < 0.05. Data are presented as mean ± SD and median [25^th^-75^th^]*.

Patient “A” had a significantly higher V_D_ at baseline measurements than patient “B”. Comparing the two patients, there was no significant difference in V_D_ following the recruitment maneuver. With patient “A,” V_D_ significantly decreased and with patient “B,” V_D_ did not significantly increase following the intervention. There was no significant alteration in end-tidal carbon dioxide (EtCO_2_) in neither of the patients following the PEEP trial (Table 2).

### Overdistension and Collapse by EIT

**Figure 4:**
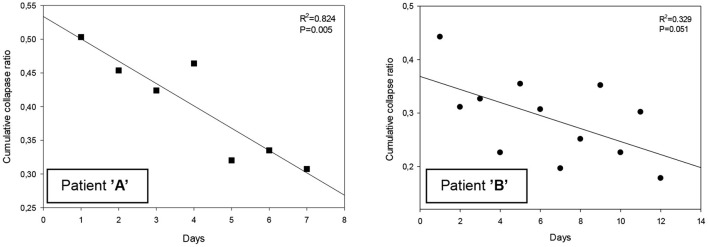
**(A,B)** Cumulative collapse ratio following PEEP trial.

**Figure 5:**
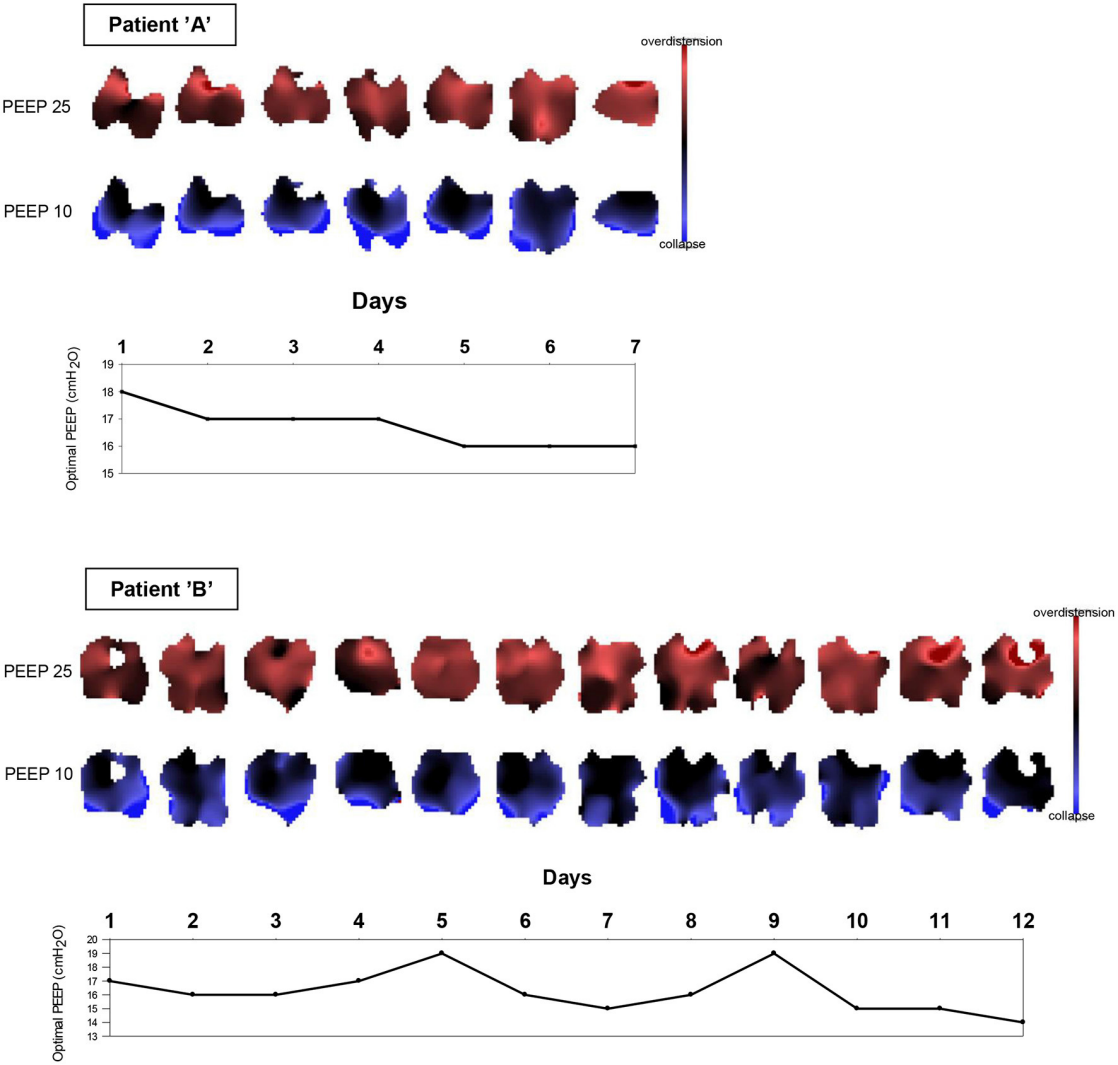
Pixel overdistension and collapse images on each day with patients **(A,B)** at top PEEP 25 and final PEEP 10 cmH_2_O and optimal PEEP levels. PEEP, positive end-expiratory pressure.

Regarding the cumulative overdistension ratio at the top-level PEEP of 25 cmH_2_O, there was no significant difference between the two subjects. Following recruitment maneuvers, patient “A” had a significantly higher cumulative collapse ratio detected by EIT than patient “B” (Table 3). With patient “A,” the cumulative collapse ratio at the end of the recruitment maneuvers revealed a significant decreasing linear regression by moving forward in days, while not with patient “B” (Figure 4)” Overdistended and collapsed regions were reconstructed at the top PEEP 25 and final PEEP 10 cmH_2_O (Figure 5). Optimal PEEP gradually decreased with patient “A” while it was fluctuating over time with patient “B” (Figure 5).

**Table 3:** Overdistension ratio at top and collapse ratio at final PEEP levels.

	**Patient “A”**	**Patient “B”**
Cumulative overdistension ratio at PEEP 25 cmH_2_O	0.37 ± 0.08	0.35 ± 0.06
Cumulative collapse ratio at final PEEP 10 cmH_2_O	0.40 ± 0.08	0.29 ± 0.08^*^

*PEEP, positive end-expiratory pressure*,

* *significant difference as compared to patient “A”, P < 0.05. Data are presented as mean ± SD*.

## Discussion

### Identification of L- and H-Phenotype

Under the investigation two severe, intubated and mechanically ventilated patients were assessed and recognized as L- and H-phenotype according to the Gattinoni classification (3). Based on the respiratory mechanics and volumetric capnography, patient “A” was revealed as L-type with low elastance, fairly normal compliance, and low V_A_/Q. On the contrary, patient “B” was identified as H-type with high elastance, low compliance, and high right-to-left shunt. On the other hand, real-time, bedside, EIT-based overdistension and collapse ratio measurements were capable to distinguish the two phenotypes. Patient “A” had a more pronounced tendency for collapse following the PEEP recruitment; while at the end of the PEEP trials, patient “B” was presented with a lower cumulative collapse ratio. Meanwhile, EIT demonstrated the transition from L- to H-type with patient “A” as the cumulative collapse ratio decreased over time at the end of the daily PEEP trials in addition to the deterioration in C_stat_.

### Respiratory Mechanics

However, the gold standard CT scan is the most valuable method to identify the pathophysiological mechanisms not just in any ARDS but also in all, severe COVID-19 pneumonia patients, assessment of respiratory mechanics serves as a surrogate (3). A simple end-inspiratory hold maneuver is capable to determine the plateau pressure and with the charted tidal volume C_stat_ can be recognized. Throughout the first days with patient “A,” an almost normal C_stat_ was revealed in line with a deep level of hypoxia. However, severe COVID-19 pneumonia meets the criteria of Berlin definition of ARDS (1), the disease acts in a very specific way which was rarely apparent in severe ARDS previously. Meanwhile, patient “B” was recognized with a considerably damaged C_stat_, the same level of grievous hypoxia was presented as with patient “A.” These observations testify the heterogeneity of COVID-19 pneumonia and the necessity of the identification of various phenotypes.

Nevertheless, L-type can transit into H-type (3, 9). This transformation was clearly demonstrable with patient “A” as C_stat_ significantly deteriorated by time cascading down to around 40 ml/cmH_2_O. As patient “A” was the same ventilated as patient “B” in pressure control mode throughout the investigation days self-inflicted lung injury (P-SILI) cannot have a share in evolving the deterioration. However, esophageal pressure measurement for estimating transpulmonary pressures was not applied. The P-SILI mechanism is much more characteristic in patients breathing spontaneously on non-invasive ventilation with an increased swing in transpulmonary pressure leading to enhance the stress at alveolar level (10). With patient “A,” the transition can be explained by the evolution of COVID-19 pneumonia on itself.

### Ventilation-to-Perfusion

In parallel with the hypothesis of V_A_/Q mismatch in L-type patients, Santamarina et al. (11) detailed the possible underlying pathomechanisms with the help of subtraction iodine mapping CT. With L-type patients, just like with our patient “A,” the low V_A_/Q is possibly secondary to loss of compensatory hypoxic pulmonary vasoconstriction leading to increased blood flow through the injured lung areas. In addition to vasoplegia around the damaged alveoli, hypoperfusion can develop in apparently healthy areas. The downregulation of angiotensin-converting enzyme 2 (ACE2) has an utmost importance in the mechanisms formerly detailed. With H-type patient “B,” the increased right-to-left shunt probably can be explained by the substantial perfusion through the atelectatic parenchyma, which is precipitated by the extended edema and the associated increase in lung weight. However, without direct lung ventilation/perfusion imaging and extravascular lung water index assessment, we could not validate our findings so they remain hypothetical.

The V_D_ measurements indicated these disturbances with both patients “A” and “B.” However, Bohr–Enghoff equation is not capable to distinguish anatomically and the alveolar dead space as it can present only their sum (12). With L-type patient “A,” a significant decrease in V_D_ was presented following the recruitment parallel to a significant decrease in PaCO_2_ and pH. Tough C_stat_ improved, one cannot exclude the possibility of development in V_A_/Q. However, this would have required an assessment of perfusion. While a significant improvement was detected in C_stat_ in both patients there was no improvement in PaO_2_/FiO_2_ which coincides with the previous observations (13, 14).

### Electric Impedance Tomography

Numerous investigations applied CT scans to assess the recruitabilty in COVID-19 pneumonia patients. However, a CT scan has an outstanding resolution; this imaging method is not suitable for serial evaluation as it requires high doses of radiation and in-hospital transportation of the critically ill. It is no far to seek that a radiation-free, bedside, real-time, functional imaging like EIT can significantly aid the follow-up in the evolution of pathophysiology (15). However, scarce literature can be hit considering the application of impedance tomography in the evaluation of COVID-19 pneumonia.

Kotani and Shono (16) followed the homogeneity of ventilation distribution by EIT following prone positioning. The investigation of Tomasino et al. (17) with ventilation distribution assessed by EIT could indicate the usefulness of prone positioning in an L-type patient. EIT can be beneficial in personalizing respiratory therapy leading to setting a higher level of PEEP in COVID-19 patients (18) than recommended by previously developed FiO_2_/PEEP tables for ARDS. Respectively, the recruitment-to-inflation ratio observed by EIT could determine recruitability with COVID-19 pneumonia (19).

Our series of PEEP trials investigated the alteration in global overdistension and collapse. During a decremental PEEP trial, the ease of the overdistended regions is characterized by an increase, while the collapse of previously open areas by a decrease in pixel compliance. For the estimation of these processes, the algorithm designed by Costa et al. (6) was applied. As expected, the ratio of overdistension was the highest at the top PEEP level in both patients. The cumulative collapse ratio was a useful tool to differentiate L-type (patient “A”) from H-type (patient “B”). Furthermore, the alteration in collapse ratio by time was capable to pursue the transition from L- to H-type with patient “A.”

L-type patient “A” had a higher tendency for collapse during the decremental PEEP phase in the first few days. Parallel with this tendency, higher optimal PEEP levels were revealed on the same days. This recognition can lead to an opposing PEEP recommendation than suggested by Gattinoni et al. (3) according to which higher PEEP settings can be advocated in L-type patients with respect to our observations, at least during the first few days following orotracheal intubation. This perception complies with the article of van der Zee et al. (18). Unambiguously with patient “B,” the H-phenotype labeling was persisting. As the condition in the ratio of overdistension and collapse was fluctuating day by day, EIT was a useful tool to individualize ventilator settings.

### Limitations

One of the main limitations of the research is the type of case series investigation. A higher number of PEEP trial instances with more COVID-19 pneumonia patients would have significantly strengthened the observations. The other limitation of the research is that a completely aerated lung was estimated following the incremental phase of the PEEP trial. With this estimation, the relative ratio of the recruitable alveolar collapse was calculated. This is limited by the time difference in EIT protocol as an absolute amount of collapse cannot be procured. However, the protocol still provides a collapse ratio related to the minimal potential collapse, but can still render information about the condition of the lungs. Comparing the EIT observations with lung CT scans performed before and after the PEEP trials would significantly promote the results. However, transferring critically ill patients for such a high radiation dose investigation on a daily basis is unethical and unfeasible. Finally, the absence of muscle paralysis could affect the lung mechanics.

## Conclusion

This is the first investigation that followed up COVID-19 pneumonia patients under EIT observation during PEEP trials on a daily basis. The estimated ratio of global collapse and overdistension defined by EIT can be a potential bedside device to differentiate L- from H-phenotype. EIT was a feasible tool to monitor the transition of L-phenotype into the other. EIT monitoring provides sufficient information about the evolution of COVID-19 pneumonia, hence promoting the daily, patient individualized settings on the mechanical ventilator. As SARS-CoV-2-associated pneumonia has a slow tendency of regression and requires long-term respiratory therapy, optimizing ventilator parameters has an utmost importance in the prevention of ventilator-associated lung injury. However, the short case number of PEEP trials and the lack of CT validation of the observations require further investigations to promote these findings.

## Data Availability Statement

The original contributions presented in the study are included in the article/supplementary material, further inquiries can be directed to the corresponding author.

## Ethics Statement

The studies involving human participants were reviewed and approved by Human Investigation Review Board of the University of Szeged. The patients/participants provided their written informed consent to participate in this study.

## Author Contributions

KM, BB, and AL conceived of the presented idea. AL carried out the experiment. TM, FH, and ÁS recorded the data. RC and SK-Z performed the numerical calculations derived by impedance tomography. KM supervised the impedance tomography calculations. TM, FH, and AL performed the statistical analysis. AL took the lead in writing the manuscript. All authors provided critical feedback about the article and contributed to the article and approved the submitted version.

## Funding

The research was supported by the German Federal Ministry of Education and Research (MOVE, Grant 13FH628IX6), by the Hungarian National Scientific Research Foundation (Grant K137995), by H2020 MSCA-RISE DCPM (Grant 872488) and by the National Research, Development, and Innovation Fund of Hungary (Grant TKP2021-EGA-02).

## Conflict of Interest

The authors declare that the research was conducted in the absence of any commercial or financial relationships that could be construed as a potential conflict of interest.

## Publisher's Note

All claims expressed in this article are solely those of the authors and do not necessarily represent those of their affiliated organizations, or those of the publisher, the editors and the reviewers. Any product that may be evaluated in this article, or claim that may be made by its manufacturer, is not guaranteed or endorsed by the publisher.
